# “From molecular to clinic”: The pivotal role of CDC42 in pathophysiology of human papilloma virus related cancers and a correlated sensitivity of afatinib

**DOI:** 10.3389/fimmu.2023.1118458

**Published:** 2023-03-01

**Authors:** Erdong Wei, Jiahua Li, Philipp Anand, Lars E. French, Adam Wattad, Benjamin Clanner-Engelshofen, Markus Reinholz

**Affiliations:** ^1^ Department of Dermatology and Allergy, University Hospital, Ludwig Maximilians University of Munich (LMU) Munich, Munich, Germany; ^2^ Dr. Phillip Frost Department of Dermatology & Cutaneous Surgery, Miller School of Medicine, University of Miami, Miami, United States

**Keywords:** HPV-related cancers, DNA methylation, scRNA, CDC42, afatinib

## Abstract

**Background:**

Human papilloma virus (HPV)-related cancers are global health challenge. Insufficient comprehension of these cancers has impeded the development of novel therapeutic interventions. Bioinformatics empowered us to investigate these cancers from new entry points.

**Methods:**

DNA methylation data of cervical squamous cell carcinoma (CESC) and anal squamous cell carcinoma (ASCC) were analyzed to identify the significantly altered pathways. Through analyses integrated with RNA sequencing data of genes in these pathways, genes with strongest correlation to the TNM staging of CESC was identified and their correlations with overall survival in patients were assessed. To find a potential promising drug, correlation analysis of gene expression levels and compound sensitivity was performed. *In vitro* experiments were conducted to validate these findings. We further performed molecular docking experiments to explain our findings.

**Results:**

Significantly altered pathways included immune, HPV infection, oxidative stress, ferroptosis and necroptosis. 10 hub genes in these pathways (PSMD11, RB1, SAE1, TAF15, TFDP1, CORO1C, JOSD1, CDC42, KPNA2 and NUP62) were identified, in which only CDC42 high expression was statistically significantly correlated with overall survival (Hazard Ratio: 1.6, *P* = 0.045). Afatinib was then screened out to be tested. *In vitro* experiments exhibited that the expression level of CDC42 was upregulated in HaCaT/A431 cells transfected with HPV E6 and E7, and the inhibitory effect of afatinib on proliferation was enhanced after transfection. CDC42-GTPase-effector interface-EGFR-afatinib was found to be a stable complex with a highest ZDOCK score of 1264.017.

**Conclusion:**

We identified CDC42 as a pivotal gene in the pathophysiology of HPV-related cancers. The upregulation of CDC42 could be a signal for afatinib treatment and the mechanism in which may be an increased affinity of EGFR to afatinib, inferred from a high stability in the quaternary complex of CDC42-GTPase-effector interface-EGFR-afatinib.

## 1 Introduction

Co-evoluted with vertebrates for more than 350 million years, the epitheliotrophic papilloma virus (PV) has already well adapted to the host tissue, the squamous epithelia of skin and mucosal surfaces (1). Despites there are numerous types of human papilloma virus (HPV) multiplying and producing progeny viruses in their hosts, most of them do not cause any detectable pathologies, if any, usually only minor and benign lesions (1).

But meanwhile, some types of HPV are responsible for approximately 30% of all cancer cases caused by infectious agents (2), including cervical squamous cell carcinoma (CESC), anal squamous cell carcinoma (ASCC) and other types of carcinoma, resulting in estimated 610,000 incident cancer cases and more than 250,000 deaths worldwide annually (3).

In terms of carcinogenesis, both the etiological subgroup of HPV and the involved anatomical location are relatively restricted. Of more than 200 types of HPV, only 15 high-risk-HPV types are identified as causes of malignant neoplasms, most prevalently HPV-16 and 18 (4). Also based on pathogenetic study in CESC, the carcinogenesis induced by HPV occurs specifically in the small, discrete cell population that localizes in the squamocolumnar junction of the cervix (5). This similar tropism and transformation of epithelial cells are also observed in the dentate line of anal canal (6), where columnar epithelium gradually transitions to squamous epithelium, suggesting a similar carcinogenetic process in ASCC (7). This most diagnosed histological type of malignant disease in anal canal (8), is developed from its precursor lesion — anal intraepithelial neoplasia (AIN), similar to the relation between CESC and cervical intraepithelial neoplasia (CIN).

Pathophysiological studies on carcinogenesis of HPV virus have demonstrated: the integration of HPV genoemes into the chromosomes will destabilize the genomic of the vulnerable host cells, inducing a secondary epigenetic re-programming (9). This process features with an overexpression of E6 and E7 genes, which could stimulate the expression and activity of DNA methyltransferase I (DNMT I), triggering a consequential hypermethylation in the host cells (10, 11). Based on these findings, it was hypothesized that, in all HPV-related cancers, the maintaining stemness-like differentiation status in epithelial cells relies on the hypermethylation induced by ongoing E6 and E7 (9), which plays an important role in the progression of cancers.

The technological developments of both profiling methods of DNA methylation and the computational approaches for processing the obtained data, have empowered us to investigate DNA methylation in different disease progressions from a global view through massive processed data, which is hard to be achieved even by dozens of “traditional” experiment-based studies (12). However, most existing related bioinformatic studies in HPV-related cancers only focused on the DNA methylation anomalies, but ignored the downstream changes. This limits the significance of the findings in these studies to some extent. Because without an integrated analysis of the transcriptomic and other downstream data, which could not only be affected by DNA methylation, but also other intricate intracellular molecular biological processes, it is hard to achieve a deep and global comprehension to the pathophysiology of HPV-related cancers.

In this study, we tried to from a more comprehensive perspective to investigate the pathophysiology of HPV-related cancers, through muti-omic bioinformatic analyses and other methodological tools. We started with analyses of DNA methylation data, integrating downstream RNA sequencing (RNA-seq) data in HPV-related cancers, aiming to identify the hub genes in the progression of HPV-related cancers. Clinical significances of these identified hub genes were then scrutinized by conducting survival analysis based on The Cancer Genome Atlas (TCGA) database, through which we sought to validate the meaning of our findings in terms of clinical prognosis. Through the above analyses, CDC42 was identified as the pivotal gene due to its significance in both pathophysiology and clinical prognosis. To further extend the implications of our findings to the treatment of HPV-related cancers, we then picked out afatinib, a selective epidermal growth factor receptor (EGFR) inhibitor, due to its most positive sensitivity correlation with CDC42 according to analyses results from Cancer Therapeutics Response Portal (CTRP). To validate these findings, we then performed *in vitro* experiments to investigate EGFR, pEGFR and CDC42 expressions, together with viabilities and proliferations in HaCaT and A431 cells transfected with HPV 16 E6 and E7 and under interventions of afatinib at different concentrations. In the final step, to make our findings theoretically self-consistent, we stated a hypothesis based on a very convincing result in Computer-Aided Molecular Docking Experiment.

Through this “molecular to clinic” research work, with a spectrum from molecular docking experiments, bioinformatic analyses in molecular biology (epigenetics and transcriptome), *in vitro* experiments of cell biology (protein expression and cell proliferation) to clilnic-associated survival analyses, we hope to shed some new light on the disease process of HPV-related cancers, lay the foundation for further developing of precise molecular targeted therapy and provide aids for clinical decision making, to better confront the challenges posed by these cancers.

## 2 Materials and methods

### 2.1 Data collection

DNA methylation data of CESC and ASCC were collected from Gene Expression Omnibus (GEO) database GSE186859, including 121 ASCC samples, 13 adjacent AIN3 samples, 9 adjacent normal samples, 9 CESC samples, 9 CIN3 samples, 10 adjacent normal cervical samples. Single-cell RNA-seq (scRNA-seq) data were collected from GEO database GSE171894 and GSE176415 (GSM5364334, GSM5364335, GSM5364336), including 2 HPV-pos. CESC samples, 2 HPV-neg. CESC samples and 3 normal samples. Bulk RNA-seq data and paired clinical information were obtained from TCGA-CESC project, excluding samples with missing clinical information or histological types other than cervical squamous cell carcinoma, and eventually 237 samples were selected for analyses.

### 2.2 Methylation profiling and data analysis

DNA methylation raw data were analyzed by the Chip Analysis Methylation Pipeline (ChAMP) R package (13). ChAMP is a comprehensive methylation analysis package, including features of quality control, identification of Differentially Methylated Probes (DMPs), Differentially Methylated Regions (DMRs), and Differentially Methylated Blocks (DMBs). Probes with detection *P*-value > 0.01, probes with <3 beads in at least 5% of samples per probe and probes located in sex chromosome were filtered out *via* champ.filter() function. Differentially designed 450K probes were normalized by function champ.norm(). Champ.DMP() function was carried out to calculate the methylation differences of p+robes. DMPs with |logFC| > 0.2 and *P*-value < 0.05 were picked.

### 2.3 Function annotation

Kyoto Encyclopedia of Genes and Genomes (KEGG) pathway analysis was performed to annotate the picked DMPs (14). FerrDb (15) and GeneCard (16) database were utilized for additional annotations. Data from GeneCard with a relevance score over median value were chosen for mapping.

### 2.4 Immune analysis

Different immune cell proportions of genes obtained from DMPs were analyzed utilizing cell type identification by calculating relative subsets of RNA transcripts (CIBERSORT) (17). Seurat R package was applied to identify genes related to different immune infiltration from CIBERSORT (18). An upper bound threshold for the percentage of mitochondrial count (5%) was defined, and the cells above the upper bound were filtered out (18). Data normalization was carried out after cell filtering that use the global-scaling normalization package LogNormalize, which divides the specific feature counts of each cell by the overall counts of that cell, divides it by 10^4^ and then performs a natural log-transformation (19). The samples were then merged into a single data set using the merge function. The FindIntegrationAnchors function was used to find the anchors, and the Inte-grateData function was used to integrate multiple data sets (20). Community detection algorithm was applied for clustering the cells, R tool FindCluster() and the parameter “resolution = 1” for controlling the number of clusters. Non-linear dimensional reduction technique Uniform Manifold Approximation and Projection (UMAP) and t-distributed stochastic neighbor embedding (t-SNE) were performed to visualize single cell clustering in low-dimension. The cluster-specific marker genes were obtained using the Findmarkers () tool in the Seurat package with default non-parametric Wilcoxon rank sum test as well as Bonferroni correction. The characteristic cellular marker reference was obtained from R package Celldex, with which cells were automatically annotated by R package SingleR (21). Plot1cell R package (22) was used to visualize and quantify the scRNA-seq data.

### 2.5 Target genes with clinical prognosis and drug selection

An RNA matrix was constructed using immune-related genes and genes annotated with specific functions obtained from the previous steps. The “WGCNA” package was used for the weighted correlation network analysis (23). This network can be used to identify highly synergistic genomes and identify candidate biomarker genes or therapeutic targets based on genomic endogeneity and genome-to-phenotype associations (23).

According to the TNM staging in WHO guideline for cervical cancer (24), in our study we defined TNM IA-IIA, which is mainly treated with surgery and has a good prognosis, as the “early group”, and TNM IIB-IV, which requires simultaneous radiotherapy and has a relative poor prognosis as the “advanced group”. By analyzing the correlation between RNA matrix and grouping information (early or advanced) in WGCNA network, gene clusters, which were most associated with cancer progression, were identified.

Data containing information on genes and weighted gene co-expression in the clusters were input into Cytoscape for visualization of network. Cytoscape molecular complex detection (MCODE) was used for finding the strongly interacting genes in the clusters (25), by setting degree cut off = 2, node score cut off = 0.2, K-core = 2, and maximum depth up to 100. The top 10 genes with the strongest interaction were filtered out and defined as hub genes.

Gene Expression Profiling Interactive Analysis 2021 (GEPIA 2021), a web-based program, was used to analyze the correlation between the expression level of the hub genes and the overall survival in CESC patients (26).

Cancer Therapeutics Response Portal (CTRP) V2, a data matrix contains profiles of chemical sensitivity, was then used to analyze the correlation between the hub gene expression levels and drug sensitivity for filtering out potentially promising drugs (27).

### 2.6 Cell culture

A431 and HaCaT were purchased from American Type Culture Collection (ATCC, Wesel, Germany). HaCaT and A431cells were cultured in DMEM (Sigma-Aldrich, Schnelldorf, Germany) media containing 10% Fetal Bovine Serum (FBS) (Sigma-Aldrich, Schnelldorf, Germany) and 1% penicillin-streptomycin (Sigma-Aldrich, Schnelldorf, Germany) at 37°C in a humidified incubator with 5% CO_2_.

### 2.7 Cell transfection

HPV16 E6 and E7 expressing plasmid as a bacterial stab (p1321 HPV16 E6 and E7, Addgene #8641) was gifted from Prof. Peter Howley (28). Sterile loops were used to steak bacterial stab on the LB agar plates, grown at 37°C in a humidified incubator with 5% CO_2_ overnight. Ampicillin-resistant colonies on LB agar plates were selected and amplified in LB/ampicillin medium overnight at 37°C in a humidified incubator with 5% CO_2_. Plasmid DNA was recovered from the bacterial culture by ethanol precipitation.

Transfection of plasmid DNA from above steps was performed with X-tremeGENE 9 DNA transfection reagent (Roche, Mannheim, Germany). X-tremeGENE 9 DNA transfection reagent was diluted with serum free DMEM to a concentration of 3 μl reagent/100 μl DMEM for a ratio of 3:1. Then, 1 μg of DNA was mixed with 100 μl diluted X-tremeGENE 9 DNA transfection reagent, and the DNA transfection reagent complex was incubated for 20 mins at RT. In 96-well plates, 5 μl DNA transfection reagent complex was added to each well and in 10-cm dishes, 500 μl was added. The cells were incubated for 24 h before further analysis.

The untransfected groups were seeded and treated at the same time and under the same conditions. The cells were cultured with the same transfection reagent complex as the transfected group, but without the addition of DNA.

### 2.8 Cell viability and proliferation assay

After 24 h transfection, cells were treated with afatinib (SML3109, Sigma-Aldrich, Schnelldorf, Germany). HaCaT and A431 cells were treated with different concentrations (0 μM, 0.1 μM, 1 μM, 10 μM) of afatinib for 24 h. Untransfected groups were treated at the same time and the same conditions. cell viability and proliferation were assessed using the Water-Soluble Tetrazolium 1 (WST-1) assay (Sigma-Aldrich, Schnelldorf, Germany). 10 µl WST-1 reagent was added to each well in 96-well plates. After incubating for 4 h at 37°C and 5% CO_2_. The absorbance of the samples at a wavelength of 440 nm was measured *via* a plate reader (Spectra MR, Dynex Technologies, Chantilly, USA).

### 2.9 Western blot

HaCaT/A431 cells were seeded on 10 cm dishes and incubated overnight, and grouping was the same as for cell viability and proliferation assays. Extraction of proteins were performed after cells were exposed to different concentrations (0 μM, 0.1 μM, 1 μM, 10 μM) of afatinib for 24 h. RIPA lysis and extraction buffer (89901, Thermo Fisher Scientific, Planegg, Germany) and protease inhibitors set (Roche, Mannheim, Germany) were used for protein extraction.

5 μl of protein ladder (Sigma-Aldrich, Schnelldorf, Germany) was used for determination of the molecular mass. 10 μl of cell lysate and 2 μl 6× loading buffer (Sigma-Aldrich, Schnelldorf, Germany) were added to each well of SDS-PAGE gel. Electrophoresis was conducted at 80 V for 50 mins, then 120 V until the marker proteins reached the bottom of gel.

PVDF membranes were activated by methanol for 5 mins. Filter Paper Sandwich (Thermo Fisher Scientific, Planegg, Germany) (sponge-filter paper-gel-membrane-filter paper-sponge) was mounted in the transfer tank and air bubbles were removed. It was transferred with 200 mA for 90 mins on ice. Membranes were blotted with 5% skim milk in for 2 h at RT. Then, the primary antibody was applied against CDC42 (HPA069590, 1:2000, Sigma-Aldrich, Schnelldorf, Germany), EGFR (AMAB90816, 1:1000, 1 µg/ml, Sigma-Aldrich, Schnelldorf, Germany), pEGFR (07-819, 1:750, Sigma-Aldrich, Schnelldorf, Germany) and GAPDH (#2118, 1:1000, Cell Signaling Technology, USA) for overnight at 4 °C. Secondary antibodies were incubated with the membranes at room temperature for 1 h. Lastly, the protein bands were captured using ChemiDoc Imaging Systems (Bio-Rad Laboratories GmbH, Feldkirchen, Germany). ImageJ were used for analysis of western blot data (29).

### 2.10 Molecular docking

Autodock Vina, a silico protein-ligand docking program, was used to examine the binding affinities and mechanisms of interaction between the drug candidate and their targets (30, 31). The molecular structure of afatinib (PubChem 10184653) was obtained from PubChem Compound (https://pubchem.ncbi.nlm.nih.gov/) (32). The 3D coordinates of CDC42 (PDB ID, 1AJE; Resolution: NA) (33) and EGFR (PDB ID, 6VH4; Resolution: 2.80 Å) (34) were downloaded from the PDB (http://www.rcsb.org/). All protein and molecular data were converted into PDBQT format for docking analysis, with all water molecules removed and polar hydrogen atoms applied. The grid box was positioned in the middle to allow for unrestricted molecular mobility and to cover the domain of each protein.

Rigid protein-protein docking (ZDOCK) was performed between CDC42 and EGFR to study the relationships (35). The PDB format of the protein structural domains were the obtained from the same database, The 3D coordinates of CDC42-GTPase-effector (PDB ID,5UPL; Resolution: 3.00 Å) interface (36) and EGFR-afatinib (PDB ID,4G5J; Resolution: 2.80 Å) (37) were downloaded from PDB. The ZDOCK module was run to identify the docking sites and calculate the ZDOCK scores.

### 2.11 Statistical analysis

The error bars in cell proliferation assays are presented as mean ± standard error, and statistical analyses for cell proliferation assays were performed using GraphPad Prism^®^ 5 (GraphPad Software, San Diego, CA, USA). Statistical analysis for DNA methylation, bulk RNA-seq data and scRNA-seq data was performed using R Statistical Software, the usage and setting of all the analysis could be found in reference of R packages (v4.2.1; R Core Team 2022).

## 3 Results

### 3.1 Methylation analysis

**Figure 1:**
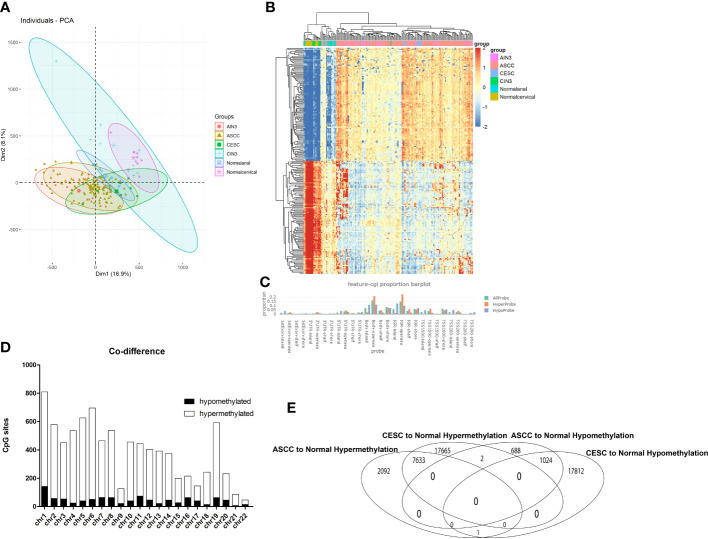
Overview of DNA methylation data. **(A)** Individuals plot of PCA, samples were represented as follows: red, AIN3. Brown, ASCC. Green, CESC. Blue cross, CIN3. Blue square, Normal cervical. Purple, Normal anal. **(B)** Heatmap of Top 200 DMPs, samples were represented as: Purple, AIN3. Red, ASCC. Blue, CESC. Green, CIN3. Light blue normal anal. Brown, normal cervical. **(C)** Proportion of the feature of CpG-islands, orange represented for hypermethylated probes, blue represented for hypomethylated probes. **(D)** The overlapped DMPs between CESC and ASCC, X axis represented for different chromosome, Y axis represented for number of DMPs, black bar represented for hypomethylated DMPs, white bar represented for hypermethylated DMPs. **(E)** Quantities of overlapped DMPs.

There were 390,065 probes that passed quality control for all subsequent analyses. Principal component analysis (PCA) was performed to compare β-values for all samples (**Figure 1A**). As shown in **Figure 1A**, no intersection between normal anal group and normal cervical group were observed, which was significantly different from that between AIN3, ASCC and CESC group, in which large proportions of intersections were observed, indicating a significant similarity of methylated sites in the disease process of AIN3, ASCC, CESC, compared to that between normal cervical and anal tissues. This similarity could also be observed in the heatmap of correlation matrix (**Figure 1B**), in which sample clusters of AIN3, ASCC or CESC could not be distinguished. Similarly, CESC samples were mixed with ASCC samples in the visualization of sample similarity based on the top 1000 most variable probes (**Supplementary Figure 1**). It could be recognized there were a large number of DMPs overlapped between AIN3, ASCC and CESC samples, while CIN3 samples were similar to normal cervical samples. Hence, we tried to analyze and compare the roles of genes between ASCC and CESC group due to the observed similarity in DMPs, which suggested similar epigenetic modifications in these two HPV-related cancers, while CIN3 and AIN3 group were excluded in this step.

As shown in **Figure 1E**, a total of 44,137 (11.31%) in 390,065 probes exhibited differing levels of methylation between CESC and normal cervical group and 11,440 (2.9%) probes showed differing levels of methylation between ASCC and normal anal group. In CESC group, compared to normal cervical group, 25300(57.4%) probes were hypermethylated while 18,837 (42.6%) probes were hypomethylated. In ASCC group, compared to normal anal group, 9726 (85.0%) probes were hypermethylated while 1714 (15.0%) probes were hypomethylated. Among these thousands of DMPs, there were only 2, that hypermethylated in CESC but hypomethylated in ASCC and only 1, that hypermethylated in ASCC but hypomethylated in CESC, indicating a very limited heterogeneity of DNA methylation in these 2 HPV-related cancers. As the final result in this step, 7633 hypermethylated DMPs and 1024 hypomethylated DMPs in both tumors were picked out for further analyses.

These DMPs varied among genomic locations, mainly enriched in open sea regions (**Figure 1C**). Besides, the distributions of both hypermethylated DMPs and hypomethylated DMPs were mostly enriched in Chromosome 1 (**Figure 1D**).

### 3.2 Function annotation

**Figure 2:**
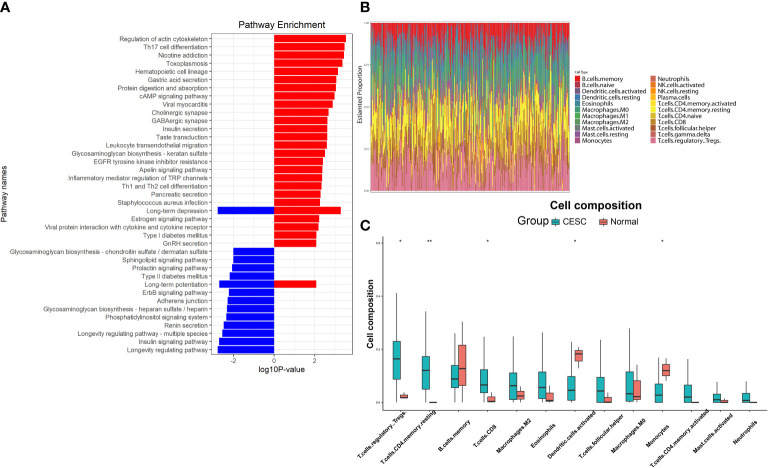
Function annotations and difference in immune infiltration of DMPs. **(A)** KEGG analysis of the DMPs, red bars represented the genes with hypermethylated DMPs, blue bars represented the genes with hypomethylated DMPs. **(B)** Immune infiltration base on the gene with DMPs, each column represented one sample, different color represented different proportions of immune cell type. **(C)** Immune cell types with significant differences in the enrichment of genes for DMPs. (*P < 0.05, **P < 0.01).

We next performed KEGG analysis on the 7633 hypermethylated DMPs and 1024 hypomethylated DMPs. **Figure 2A** showed the regulatory orientations of DMPs in the genes and the functions of these genes. Red bars represented genes with hypermethylated DMPs (*P* < 0.05) and blue bars represented genes with hypomethylated DMPs (*P* < 0.05). Most of these genes were associated with carcinogenesis and immune. In addition, HPV infection pathway (173/3314), oxidative stress (15/3314), ferroptosis (17/3314) and necroptosis (48/3314) were also enriched. Existing evidences showed that oxidative stress, ferroptosis and necroptosis processes are closely related to HPV infection and could occur in immune cells and epithelial cells (38–40). Hence, we supposed these enrichments in our study could be a result of HPV infection and subsequential HPV-induced carcinogenesis. Therefore, genes annotated with these functions were also chosen for further analyses.

Considering that KEGG is a relatively broad mapping database, there might be omissions in details. For example, only markers of genes are compared in the mapping of ferroptosis, without any inducer, promoter and driver included. After further annotation, 264 genes were identified as oxidative stress-associated, 23 genes were ferroptosis-associated and 65 genes were necroptosis-associated. CIBERSORT enrichment analysis for genes with overlapped methylations (**Figure 2B**) showed immune cell proportions mainly different in T-reg cells, CD4+ T cells, CD8+ T cells, dendritic cells and monocytes (**Figure 2C**). This result derived from CESC data due to the current absence of RNA-seq data and clinic data in ASCC.

### 3.3 Immune analysis

Immune differences were obtained from CIBERSORT analysis. However, the alterations of immune markers were associated with large-scale genetic alterations in immune infiltrations. In order to have a more precise understanding of immune alterations, we further obtained differential immune infiltration information by analyzing genes in DMPs *via* CIBERSORT. Then genes regarding these immune alterations were identified from scRNA-seq data.

**Figure 3:**
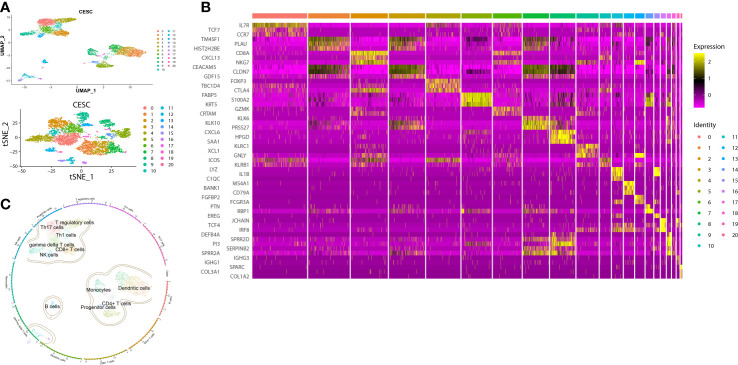
Clusters, marker genes and annotations of CESC scRNA-seq data. **(A)** The dimension reduction of CESC scRNA-seq. Visualization of separate clusters based on UAMP and tSNE. **(B)** Heatmap of the most significant marker genes, each cluster showed top 3 marker genes. **(C)** Clusters annotated by immune cell markers.

To analyze the immune identities and functions of the cells, we first clustered and visualized the cells based on the scRNA-seq data. CESC scRNA-seq were divided into 21 clusters according to the Seurat FindCluster() function, the UMAP and tSNE algorithm from the Seurat R package (**Figure 3A**). 10,959 marker genes were obtained from 21 clusters based on the Findmarker() function. Top 3 marker genes of each cluster were used for heatmap visualization. The different abundances of marker gene expression were used for further analysis (**Figure 3B**). Reference data of cellular markers were obtained from Celldex, with which SingleR could annotate clusters automatically (**Figure 3C**). Clusters with cell type annotation as T-_reg_ cells (Cluster 4, marker: FOXP3), CD4^+^ T cells (Clusters 5, 7, 18, marker: IL7R), CD8^+^ T cells (Cluster 2, marker: CD8A), dendritic cells (Cluster 11, 13, 16, marker: LYZ) and monocytes (Cluster 8, 17, marker: CXCL6) were selected as targets.

### 3.4 Target genes with clinical prognosis and drug selection

According to the selected target clusters in the previous step, 7722 immune-associated genes were obtained from scRNA-seq data, Together with genes in HPV infection pathway, oxidative stress-associated genes, ferroptosis-associated genes and necroptosis-associated genes, a new matrix including expression profiling data and clinical data was built.

**Figure 4:**
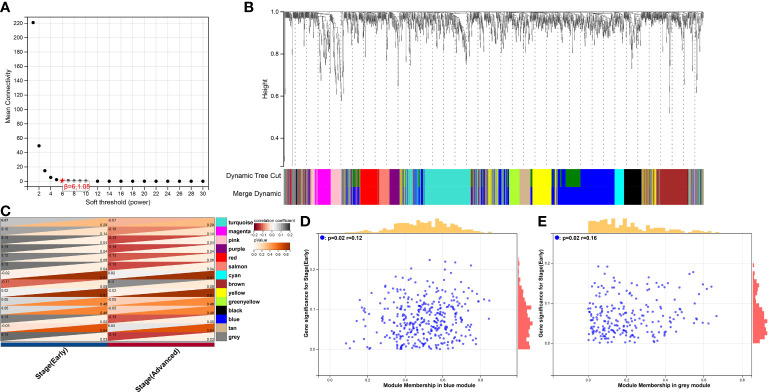
WGCNA analysis of the correlation between gene modules and clinical features. **(A)** The mean connectivity and scale-free topology index for each power value between 1 to 20. Investigation of the mean connectivity (degree, Y axis) for different soft-thresholding powers (X axis). **(B)** Dendrogram of genes in new matrix (Associated to ferroptosis, necroptosis, oxidative stress, HPV infection pathway, T-reg cells, CD4^+^ T cells, CD8^+^ T cells, dendritic cells and monocytes) clustered based on a dissimilarity measure (1-TOM). Densely linked, highly co-expressed genes are grouped together on the dendrogram’s branches. **(C)** Correlations of modules and clinical feature. Each row corresponded to a module, The number in the upper left corner represents the correlation, and the number in the lower right corner represents the *P*-value. **(D)** Scatter plot of module membership (MM) vs. gene significance (GS) in blue modules. MM presents the correlation between gene expression and each module eigengene. GS represents the association between gene expression and each trait. In both modules, GS and MM have a high correlation. **(E)** Scatter plot of module membership (MM) vs. gene significance (GS) in grey modules.

The development of the WGCNA scale-free co-expression network allowed the identification of the correlations between genetic characteristics and clinical features. Co-expression network was constructed using the new matrix. We used pickSoftThreshold function to select soft threshold power β = 6 which ensured a scale-free network (**Figure 4A**). Then, 14 distinct gene modules were generated based on hierarchical clustering dendrogram (**Figure 4B**). Previously defined clinical features “Early group” and “Advanced group” were input. Pink module (*r* = -0.13, *P* = 0.04), purple module (*r* = -0.14, *P* = 0.04), salmon module (*r* = -0.13, *P* = 0.04), blue module (*r* = -0.13, *P* = 0.05) and grey module (*r* = -0.14, *P* = 0.03) showed significant correlations with the progression of cancer (**Figure 4C**). In order to assess the probable biological function of these modules, correlations between gene significance (GS) and module membership (MM) were evaluated. These correlations were shown in the form of scatter plots (**Figures 4D, E**), in which the modules had demonstrated a significant association between GS and MM, indicating that the genes in those modules are not only co-expressed but also positively linked to clinical features. To ensure the integrity of these results, all modules associated with clinical features were included in the study rather than just the most significant ones.

**Figure 5:**
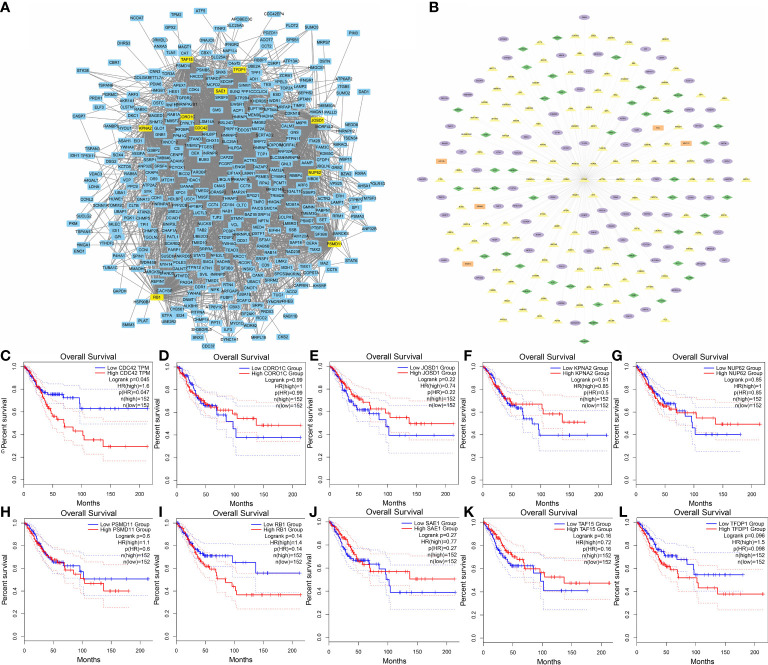
Identification of hub genes. **(A)** Top 10 MCODE scoring genes were highlighted in the co-expression network. **(B)** Hub gene CDC42 with its highly co-expressed neighbors. Green diamonds represented genes associated with ferroptosis, purple ellipses represented genes clustered in immune cell clusters of scRNA, orange rectangles represented genes related to necroptosis, and yellow triangles represented genes annotated with oxidative stress. **(C)** KM curves (Kaplan–Meier estimator) showed the correlation between CESC overall survival and CDC42 expression. **(D)** KM curves showed the correlation between CESC overall survival and CORO1C expression. **(E)** KM curves showed the correlation between CESC overall survival and JOSD1expression. **(F)** KM curves showed the correlation between CESC overall survival and KPNA2 expression. **(G)** KM curves showed the correlation between CESC overall survival and NUP62 expression. **(H)** KM curves showed the correlation between CESC overall survival and PSMD11 expression. **(I)** KM curves showed the correlation between CESC overall survival and RB1 expression. **(J)** KM curves showed the correlation between CESC overall survival and SAE1 expression. **(K)** KM curves showed the correlation between CESC overall survival and TAF15 expression. **(L)** KM curves showed the correlation between CESC overall survival and TFDP1 expression.

Cytoscape plugin MCODE was performed to find the highly interconnected regions in the network of all the nodes and edges. A node with more interconnected neighbors could achieve a higher score. In the highest region detected by MCODE, the 10 highest scoring genes were selected as hub genes (**Figure 5A**) (**Supplementary Table 1**), including PSMD11, RB1, SAE1, TAF15, TFDP1, CORO1C, JOSD1, CDC42, KPNA2 and NUP62. Next, the correlations between expressions of these gene and survival in CESC patients were analyzed *via* GEPIA2021 online tool (**Figures 5C–L**). The results showed all the hub genes manifesting correlations with cancer progression to some extent. However, only the correlation in CDC42 was statistically significant with the survival of CESC patients (Hazard Ratio: 1.6, *P* = 0.045), which implied patients with higher expression level of CDC42 had worse prognosis. Hence, we picked CDC42 as the target for downstream studies due to its significance in both pathophysiological and clinical levels. All the connections of CDC42 were listed in network (**Figure 5B**), green diamonds represented genes associated with ferroptosis, purple ellipses represented genes clustered in immune cell clusters of scRNA, orange rectangles represented genes related to necroptosis, and yellow triangles represented genes annotated with oxidative stress.

**Figure 6:**
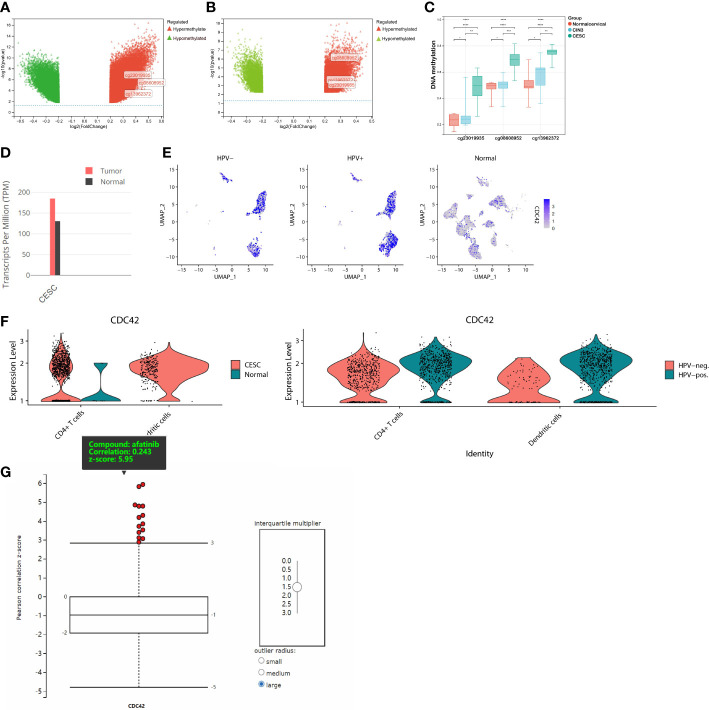
DNA Methylation, RNA expression, scRNA expression and chemical compounds sensitivity of CDC42. **(A)** Volcano plot of the DMPs between CESC and normal samples. Red plot corresponded to hypermethylated probe, green plot corresponded to hypomethylated probes. **(B)** Volcano plot of the DMPs between CESC and CIN3 samples. **(C)** β value of CDC42 DMPs (cg08608952, cg13962372 and cg23019935) in different groups. **(D)** RNA expression of CDC42 in CESC and normal samples. **(E)** Colors single cells on a dimensional reduction plot according to the expression of CDC42. **(F)** Quantifies of CDC42 in CD4^+^ T cells and dendritic cells between CESC and normal samples, HPV-pos. CESC and HPV-neg. CESC. **(G)** Correlation analysis of CDC42 expression level and chemical compounds sensitives, afatinib showed the significantly and positively correlation. *P<0.05, **P<0.01, ***P<0.001, ****P<0.0001.

Before investigating the potential role of CDC42 in therapeutic development, it was necessary to understand the alteration of CDC42 during HPV infection and carcinogenesis. There were 3 DMPs in CDC42, cg08608952, cg13962372 and cg23019935. Volcano plots showed the different DNA methylation of CDC42 between CESC and normal cervical group (**Figure 6A**), CESC and CIN3 group (**Figure 6B**). Compared to the normal cervical group, hypermethylation of these 3 DMPs was significant in CESC group, while slightly in CIN3 (**Figure 6C**). In the RNA profiling, the expression of CDC42 was higher in CESC than normal cervical group (**Figure 6D**). This result could also be observed from scRNA-seq data. Compared to normal samples, CDC42 was upregulated in CESC samples (**Figure 6E**), implying that the upregulation of CDC42 may be driven by carcinogenesis. To figure out the impact of HPV infection on CDC42, quantifies of CDC42 were performed between HPV-pos CESC, HPV-neg CESC and normal group (**Figure 6F**). In the CESC scRNA-seq data, CDC42 was grouped in cluster 1 (Dendritic cells), cluster 3 (Dendritic cells) and cluster 7 (CD4^+^ T cells). Violin plots showed the expression of CDC42 in dendritic cells and CD4^+^ T cells between 3 groups. Compared with CESC, CDC42 expression was lower in normal tissues. Whereas in comparison among CESC samples, the expression was higher in the HPV-pos group than in the HPV-neg group, though not significantly. Hence, we considered the upregulation of CDC42 was not only caused by carcinogenesis, but also HPV infection.

As of now, through bioinformatic analyses, we had partly understood the underlying driving factors of CDC42 upregulation in HPV-related cancer and its impact on the prognosis of CESC patients, but the potential contribution of these findings to clinical therapeutic development remained unclear. A correlation analysis between CDC42 expression level and chemical compound sensitivities was performed *via* CTRP database. The result explained that the expression of CDC42 was significantly and positively correlated with 14 chemical compounds, among which EGFR inhibitor afatinib was the most significant one (**Figure 6G**). To validate these findings in the analyses above, we performed *in vitro* experiments to assess the alterations of cell proliferation, cell viability and protein expression.

### 3.5 Cell viability and proliferation

**Figure 7:**
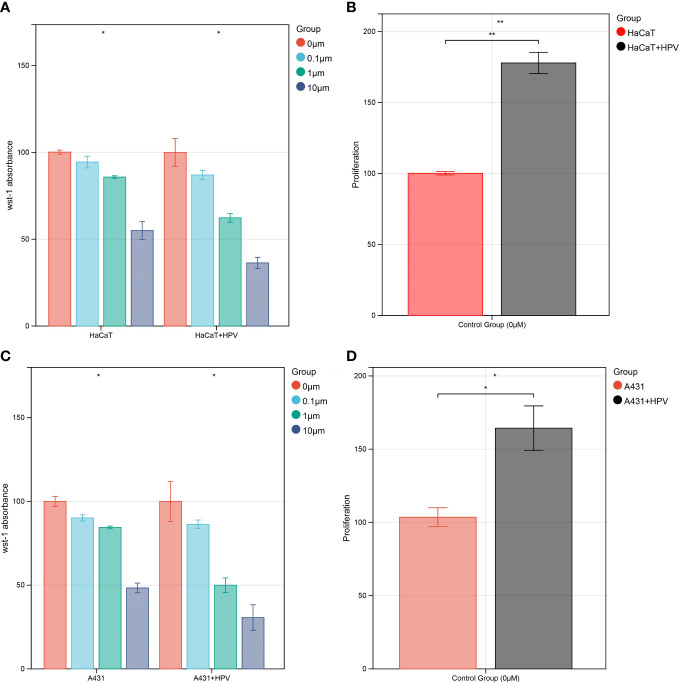
*In vitro* validation of CDC42 function. **(A)** WST-1 cell viability assay of HaCaT cell, 0 μM afatinib groups were set as 100%. **(B)** WST-1 cell proliferation test of HaCaT cells transfected with HPV E6 and E7, untransfected and untreated HaCaT cells groups were set as 100%. **(C)** WST-1 cell viability assay of A431 cell, 0 μM afatinib groups were set as 100%. **(D)** WST-1 cell proliferation test of A431 cells transfected with HPV E6 and E7, untransfected and untreated A431 cells groups were set as 100%. *P<0.05, **P<0.01.

WST-1 assays were carried out on HaCaT/A431 cells with or without HPV16 E6 and E7 transfection in the presence of various concentrations (0 μM, 0.1 μM, 1 μM, and 10 μM) of afatinib in order to assess the changes in the viability and proliferation of cells. In each group, the spectrophotometric readings of cells without exposure to afatinib were used as the relative reference standard in the figure (100%) (**Figures 7A, C**). Similarly, to compare cell proliferations between HaCaT/A431 cells with or without HPV transfection, the spectrophotometric readings of cells without transfection and exposure to afatinib were set as relative reference standard (100%) (**Figures 7B, D**). Cell proliferations of HaCaT and A431 were increased after transfected with HPV16 E6 and E7 (*P* < 0.05) (**Figures 7B, D**). In HaCaT cells, afatinib was unable to reach the half-maximal inhibitory concentration (IC50) at 10 μM, while in HaCaT cells transfected with HPV had a lower IC50 at 1 to 10 μM (**Figure 7A**). Similar results could be observed in A431 cells. In A431 cells without transfection, IC50 of afatinib was between 1-10 μM and a lower IC50 was observed after transfected with HPV16 E6 and E7 (**Figure 7C**).

### 3.6 Western blot

Cell proliferation and cell viability assays confirmed that transfection with HPV16 E6 and E7 can make cells more sensitive to afatinib. However, the alterations in protein-level in this process remained unclear. A431 and HaCaT cells were treated as described above. Protein expressions were measured after intervention of afatinib for 24 h (sc. 48 h after transfection, **Supplementary Figure 2A**). Transfection with HPV16 E6 and E7 enhanced the effect of afatinib. Quantitative analysis through Image J showed that, compared to group without HPV transfection, lower expression of pEGFR in the HPV transfected group at the same afatinib concentration could be observed, accompanied by an upregulation of CDC42 (**Supplementary Figure 2B**). This result should be interpreted as a general trend but not a precise quantification. Because we did not expect a clear mechanistic interpretation only through Western Blot and without further experiments. We aimed only to observe the trend, so no replicate experiments were performed and the results were thus not statistically significant. For this reason, WB results are placed in the supplementary material.

### 3.7 Molecular docking

**Figure 8:**
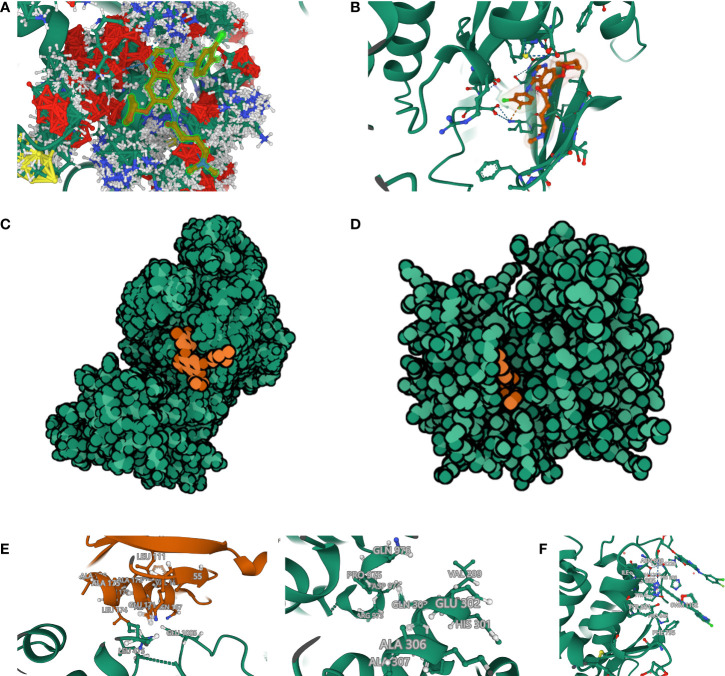
Molecular docking and rigid protein–protein docking. **(A)** Binding mode of afatinib to CDC42. CDC42 was set as ball-and-stick model with gaussian volume. The amino acids involved in the interaction are shown in a ball-and-stick model, the amino acids did not involve in the interaction were showed as cartoon. **(B)** Binding mode of afatinib to EGFR. **(C)** The Molecule of the Month feature used cartoon illustrations to demonstrate the overlay of the crystal structures of afatinib and CDC42. **(D)** The Molecule of the Month feature used cartoon illustrations to demonstrate the overlay of the crystal structures of afatinib and EGFR. **(E)** CDC42 formed hydrogen bonds with the extended GTPase-effector interface amino acid sites. **(F)** Extended GTPase-effector interface formed hydrogen bonds with EGFR amino acid sites. **(G)** Binding mode of afatinib to CDC42- Extended GTPase-effector interface -EGFR.

Afatinib acts as a targeted inhibitor of EGFR but has a high positive sensitivity correlation with CDC42 expression. We initially hypothesized that CDC42 also has a high affinity with afatinib and therefore performed molecular docking analysis. Using Autodock Vina, the binding poses and interactions of afatinib with CDC42 and EGFR were acquired. Binding energy was calculated for each interaction (**Figures 8A, B**). Results showed that afatinib bound to CDC42 and EGFR through *via* apparent hydrogen bonds and strong electrostatic interactions. Furthermore, afatinib successfully occupied the hydrophobic pockets of CDC42 and EGFR (**Figures 8C, D**).

Binding energy <-7.0 kcal/mol indicates strong binding activity of ligand to receptor. There are 9 binding models of afatinib to EGFR and all the binding models had low binding energy (-9.186 kcal/mol, -9.063 kcal/mol, -8.639 kcal/mol, -8.601 kcal/mol, -8.365 kcal/mol, -8.296 kcal/mol, -8.216 kcal/mol, -8.148 kcal/mol and -8.027 kcal/mol), indicating a highly stable binding between EGFR and afatinib. The only binding models of afatinib to CDC42 had a low binding energy of -81.793 kcal/mol, which means the affinity of CDC42 to afatinib even higher than EGFR. The process of ligand binding to proteins is very complex. In addition to the binding energy, the evaluation of affinity also requires the formation of two hydrogen bonds with hinge when the small molecule binds to the protein. In **Figures 8A–G**, the dashed line represents interaction force, and amino acids involved in the interaction are shown in a ball-and-stick model, in which afatinib could be found to have an intensive interaction with CDC42.

The affinities of afatinib to both CDC42 and EGFR were confirmed. However, it is still unclear how CDC42 acts on EGFR after binding to afatinib. We tried to simulate and calculate the CDC42-afatinib-EGFR interactions, but no interaction could be found. In the PDB database, we found that CDC42 could be bound by extended GTPase-effector interface. Hence, we tried to construct the complex of CDC42-GTPase-effector interface-EGFR-afatinib. ZDOCK provides docking of protein structures, and the higher the ZDOCK score, the stronger the docking. The top 10 best ZDOCK score of CDC42-GTPase-effector interface-EGFR-afatinib were 1264.017, 1255.494, 1219.203, 1187.345, 1156.167, 1155.194, 1154.395, 1144.608, 1134.683 and 1114.190. As shown in figure (**Figure 8E**), CDC42 could form hydrogen bond links with amino acid sites such as GLU 171, ASN167, LEU165, VAL 168, PHE 169 and LYS 166-GTPase-effector interface GLU1005, ARG 489, LEU 475. GTPase-effector interface formed hydrogen bond links with amino acid sites such as GLN303, ASN996-PRO 975, ALA 972 EGFR (**Figure 8F**). EGFR binds to afatinib through amino acids such as ASN 808, HIS 988, PRO 848 forming hydrogen bonds and strong interactions (**Figure 8G**).

Comprehensive analysis revealed that CDC42- GTPase-effector interface-EGFR-afatinib formed a stable docking model.

## 4 Discussion

Despite the global rollout of HPV vaccines, HPV-related cancers still cause huge health crisis especially in developing countries. Cervical cancer (80% histological type is CESC) remains the 4th most common malignancy in women and one of the leading causes of death in women diagnosed with cancers (41). The overall ASCC incidence increased 2.7% and incidence-based mortality increased 1.9% annually from 2001 to 2015 (42), and the 5-year survival rate of patients with metastases is only 32% (43). In terms of molecular targeted treatments, as commented in an article, the very limited number of clinical trials for CESC showed “encouraging but limited” effects on survival of patients (44). This is partially restricted by our understanding to the pathophysiology in HPV-related cancers. Phosphatidylinositide 3-kinases (PI3K) pathway is the most investigated pathway in CESC, but it has been proven difficult to design molecular targeted therapies based on this pathway (41). For metastatic ASCC, up to now, the only molecular targeted drug entering the clinical trial is cetuximab, coincidentally, also an EGFR blockade. But the combination of cetuximab with conventional chemoradiation showed severe adverse effects, resulting in trials closures (45, 46). For HPV-related cancers, novel molecular targeted drugs with promising efficacy and safety are still waited to be developed. In our previous study (47), we have found a resemblance of the prognostic effect of hub genes in CESC and head and neck squamous cell carcinoma (HNSCC), suggesting similar intracellular alterations in the HPV-related cancers, which we aimed to further investigate in this study. Through these studies, we hope to facilitate the development of novel molecular targeted drugs for HPV-related cancers.

The development of novel molecular targeted drugs relies on comprehension of pathophysiology in cancers. Despite considerable technological advances in the detection and analysis of DNA methylation, which allowed us to study the pathophysiology of HPV-related cancers from a new entry point, existing relevant studies have mostly focused only on epigenetic modifications but ignored the alterations in other levels, such as transcription. This limited the strength of findings, because some significant findings derived from DNA methylation data could have no similar significance in the level of transcription or protein expression.

Aiming to make our findings more significant, we conducted analyses based on integrated multi-omic data. Based on the analyses of DMPs from DNA methylation data in ASCC and CESC, we confirmed a high consistency of epigenetic modifications in these two HPV-related cancers (**Figure 1**). Through function annotations (**Figure 2**) of aberrant methylated gene, we identified the significantly altered pathways (immune, HPV infection, oxidative stress, ferroptosis and necroptosis) in HPV-related cancers. In terms of immune cell infiltration, CESC tumor tissue showed obvious immunosuppression, specifically manifested as a significant increase in T-reg cells and a significant decrease in activated dendritic cells (**Figure 2C**). Integrating with RNA-seq data in these pathways, we then analyzed the correlations of genes in these pathways with TNM staging of CESC through WGCNA scale-free co-expression network (**Figure 4**). In this step, 10 hub genes (PSMD11, RB1, SAE1, TAF15, TFDP1, CORO1C, JOSD1, CDC42, KPNA2 and NUP62) were identified, in which only the expression level of CDC42 was statistically significant in the correlation with overall survival in CESC patients (**Figure 5**). In the next step, we tried to further investigated the role of CDC42 in the pathophysiology of HPV-related cancer, including investigating the DMPs of CDC42 (**Figure 6C**), the difference of CDC42 expression in tumor and normal tissue (**Figure 6D**), in HPV-pos and HPV-neg samples (**Figures 6E, F**), respectively. In this step, we observed an upregulation of CDC42 in the CESC and HPV-pos CESC group. Based on the findings above, with various methods and from several aspects, we observed a significance of CDC42 in the pathophysiology of HPV-related cancers. Taking this as a starting point, next we tried to explore the potential implication of CDC42 in the design of molecular targeted therapy. Afatinib was picked up in this step due to its most significant positive correlation in sensitivity with the expression level of CDC42 (**Figure 6G**). *In vitro* experiments have been performed to validate these findings. Based on the results of Western blot, an upregulated expression of CDC42 was observed in A431 cells compared with HaCaT cells and in cells transfected with HPV E6 and E7 compared with those without HPV transfection (**Figure 7E**). Based on the results of WST-1 assays, the inhibitory effect of afatinib on proliferation and viability of A431 cells was confirmed, especially enhanced in cells transfected with HPV E6 and E7 (**Figures 7A–D**). Out of curiosity in the exact molecular interaction mechanism of CDC42 and afatinib, we further performed molecular docking experiments, through which an extremely stable CDC42-GTPase-effector interface-EGFR-afatinib complex was found (**Figure 8**), Inspired by this finding, we hypothesized that through this complex, CDC42 could increase the affinity of EGFR to afatinib, leading to a positive correlation of the expression level of CDC42 with the sensitivity of afatinib.

CDC42 is a member of the small GTPase family and plays a role in epithelial to mesenchymal transition, angiogenesis, cell cycle progression, oncogenic transformation, migration/invasion and tumor growth (48). Similar to the results of bioinformatic analyses in our study, based on immunohistochemistry of 162 CESC samples, Ma et al. had observed an up-regulated expression of CDC42 in protein level and a correlated progression in clinical stage (49). In the subsequent study, the same research group had reported a significantly higher expression of CDC42 in HeLa cells than control cells and an increased migration ability of HeLa cells after being transfected with CDC42 plasmids, which may be derived from an improved pseudopodia formation (50). The finding of high CDC42 expression in CESC-derived HeLa cells is consistent with our findings in HPV-transfected skin squamous cell carcinoma-derived A431 cells, indicating a commonality of CDC42 alteration in HPV-related cancers [HPV18 transcript in HeLa cells discovered by Prof. Hausen in 1985 had made HeLa cells not only the first immortal human cell line, but also the first HPV-related cancers cell line (51)].

Unfortunately, in spite of being involved in multiple important processes in cancer progression, CDC42 is hard to be targeted with a specific inhibitor, due to its high homology within the other Rho family GTPases and in the wider Ras superfamily (52). However, in accordance with our findings, many studies have demonstrated the associations between CDC42 and EGFR, which could be considered as an alternative pathway of action. A study reported CDC42 bound with coatomer protein complex (γCOP) could induce the accumulation of EGFR in cells. In addition, an overexpression of CDC42 could also inhibit the degradation of EGFR, inducing an increased level of EGFR, which could lead to cancer progression (48).

Afatinib is mainly used to treat cases of non-small cell lung cancer (NSCLC) that harbor mutations in the EGFR (53). But in HPV-related cancers its role was seldom investigated and the clinical evidence is very limited. A case report showed, after administrated with afatinib as a single agent for 1 month, an EGFR-amplified metastatic CESC patient achieved a partial response (PR), with a significant lesion shrinkage observed (54). In our study, we supposed and verified that the CDC42 upregulation can be considered as a signal for afatinib treatment in HPV-related cancers. Further efforts should be made including conducting validation in *in vivo* models.

There are also limitations in our study. Although we had identified the 3 DMPs in CDC42 (cg08608952, cg13962372 and cg23019935), we could not clearly interpret their roles in the regulation of transcription of CDC42. Moreover, due to the lack of relevant suitable data in other HPV-related cancers, such as HNSCC, the findings in our study are specific to ASCC or CESC, and further validation is needed in other HPV-related cancers.

In conclusion, we have identified CDC42 as a pivotal gene in the pathophysiology of HPV-related cancers. The upregulation of CDC42 could be a signal for afatinib treatment and the mechanism in which is probably an increased affinity of EGFR to afatinib, inferred from a great stability in the complex of CDC42-GTPase-effector interface-EGFR-afatinib. Through these findings, we hope to provided new insights into the disease mechanism of HPV-related cancers and lay the foundation for afatinib as a potential promising molecularly targeted drug for these cancers.

## Data availability statement

The datasets presented in this study can be found in online repositories. The names of the repository/repositories and accession number(s) can be found in the article/**Supplementary Material**.

## Author contributions

LF, BC-E and MR conceived and designed the study. EW and JL analyzed the data and drafted the manuscript. EW and PA performed the experiments. EW prepared the figures. AW polished and revised the manuscript. All authors contributed to the article and approved the submitted version.
