# Supplementary material for: “From molecular to clinic”: The pivotal role of CDC42 in pathophysiology of human papilloma virus related cancers and a correlated sensitivity of afatinib
Source: Front Immunol. 2023 Mar 1;14:1118458. doi: 10.3389/fimmu.2023.1118458 (PMC10014535; doi:10.3389/fimmu.2023.1118458)
Supplement: Supplementary file 1 [file DataSheet_1.docx]

Supplementary Material


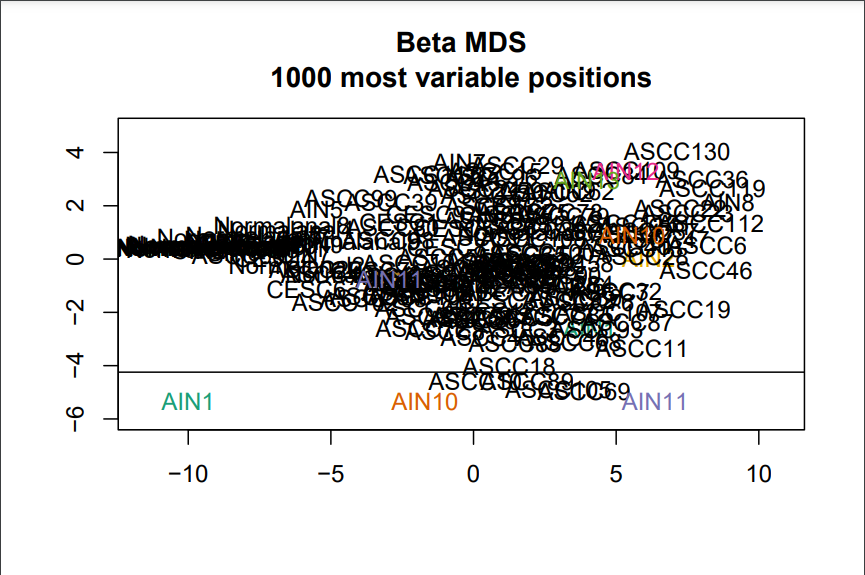


**Supplementary Figure 1. Homogeneity of samples and their grouping into diseased groups.**

The homogeneity of samples and their grouping into diseased groups were shown using multi-dimensional scaling (MDS) based on 1000 most variable points.


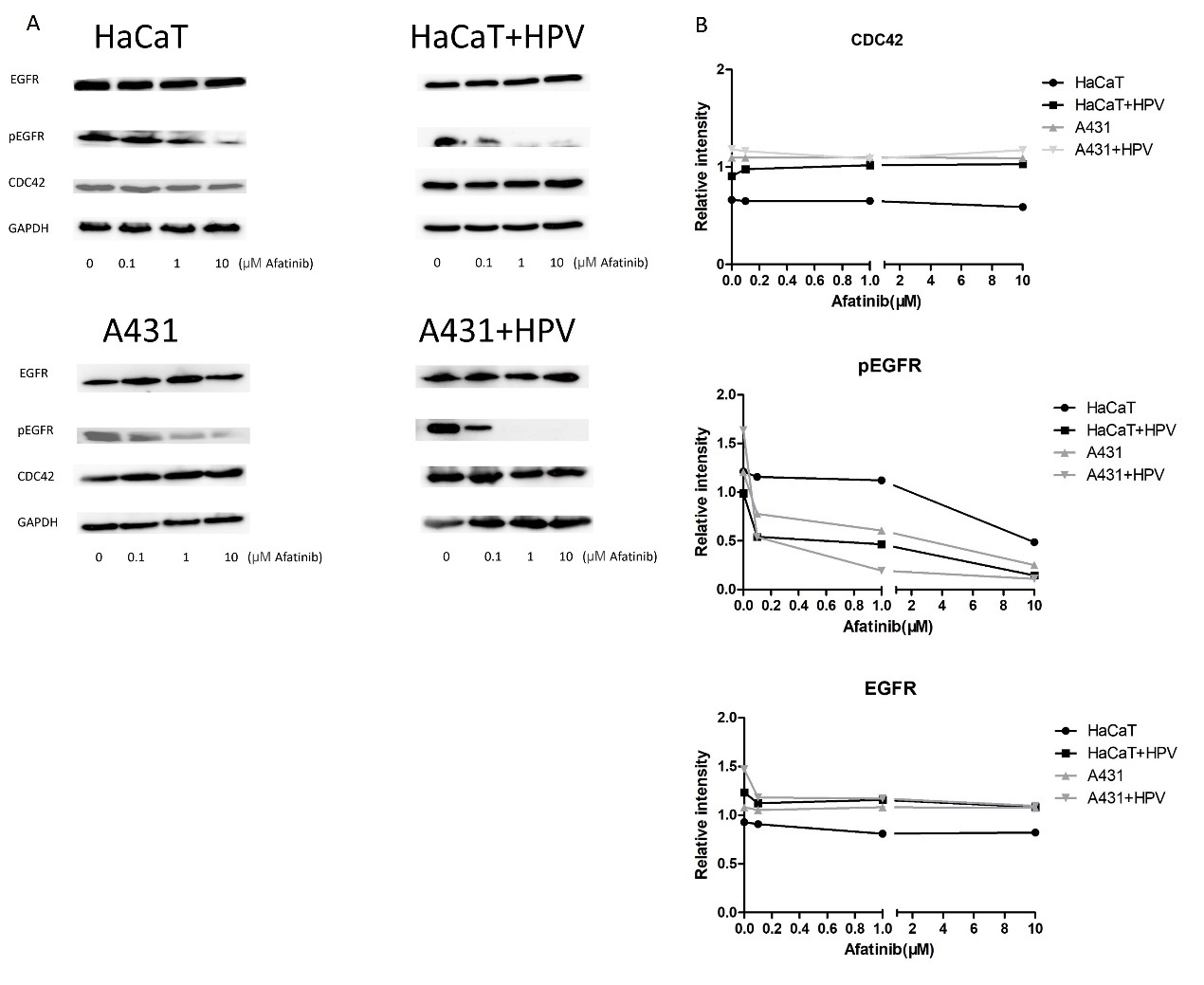


**Supplementary Figure 2. Western blot results.**

A. Western blot results of EGFR, pEGFR and CDC42.

B. Quantitative analysis of the western blot results. Experiment was performed only once, so there was not statistical analysis.

**Supplementary Table 1.** Genes with top 10 MCODE scores

| Name | MCODE Score |
| --- | --- |
| NUP62 | 161.4809 |
| JOSD1 | 160.7336 |
| KPNA2 | 160.5185 |
| TAF15 | 159.1914 |
| SAE1 | 158.8171 |
| PSMD11 | 158.6074 |
| TFDP1 | 158.0507 |
| CORO1C | 157.5923 |
| CDC42 | 157.4694 |
| RB1 | 157.3383 |
